# What are the digestion and absorption models used to reproduce gastrointestinal protein processes? A protocol for systematic review

**DOI:** 10.1097/MD.0000000000026697

**Published:** 2021-07-30

**Authors:** Anna Beatriz Santana Luz, Rafael Oliveira de Araújo Costa, Gidyenne Christine Bandeira Silva de Medeiros, Grasiela Piuvezam, Thais Souza Passos, Ana Heloneida de Araújo Morais

**Affiliations:** a Biochemistry and Molecular Biology Postgraduate Program, Biosciences Center, Federal University of Rio Grande do Norte, Natal, RN, Brazil; b Department of Nutrition, Center for Health Sciences, Federal University of Rio Grande do Norte, Natal, RN, Brazil; c Public Health Postgraduate Program, Center for Health Sciences, Federal University of Rio Grande do Norte, Natal, RN, Brazil; d Department of Public Health, Center for Health Sciences, Federal University of Rio Grande do Norte, Natal, RN, Brazil; e Nutrition Postgraduate Program, Center for Health Sciences, Federal University of Rio Grande do Norte, Natal, RN, Brazil.

**Keywords:** animals, cells, gastrointestinal tract, in vitro techniques, peptides, protocols

## Abstract

**Background:**

Animal, cell, and in vitro studies have been applied to simulate the human gastrointestinal tract (GIT) and evaluate the behavior of biomolecules. Understanding the peptides and/or proteins stability when exposed to these physiological conditions of the GIT can assist in the application of these molecules in the treatment of diseases such as obesity. This study describes a protocol of systematic reviews to analyze the methodologies that mimic the digestive and absorptive processes of peptides and/or proteins.

**Methods:**

The protocol follows the guidelines described by Preferred Reporting Items for Systematic Reviews and Meta-Analyzes Protocols (PRISMA-P). The search strategies will be applied in the electronic databases PubMed, ScienceDirect, Scopus, Web of Science, Evidence portal, Virtual Health Library, and EMBASE. The intervention group will be formed by in vivo, in cells, and in vitro (gastrointestinal simulating fluids) studies of digestion and absorption of peptides and/or proteins presenting a schedule, duration, frequency, dosages administered, concentration, and temperature, and the control group consisting in studies without peptides and/or proteins. The selection of studies, data extraction, and assessment of the risk of bias will be carried out independently by 2 reviewers. For animal studies, the risk of bias will be assessed by the instrument of the Systematic Review Center for Experimentation with Laboratory Animals (SYRCLE) and the Office of Health Assessment and Translation (OHAT) tool will be used to assess the risk of bias in cell studies.

**Results:**

This protocol contemplates the development of 2 systematic reviews and will assist the scientific community in identifying methods related to the digestive and absorptive processes of peptides and/or proteins.

**Conclusion:**

Both systematic reviews resulting from this protocol will provide subsidies for the construction of research related to the clinical application of bioactive peptides and/or proteins. In this context, they will make it possible to understand the gastrointestinal processes during administering these molecules, as the gastrointestinal environment can affect its functionality. Therefore, validating the effectiveness of these protocols is important, as it mimics in vitro biological conditions, reducing the use of animals, being consistent with the reduction, refine and replace program.

## 1 Introduction

Several bioactive molecules have emerged as candidates for clinical application in treating of obesity and associated diseases, such as bioactive proteins from animals and vegetables, phenolic acids, anthocyanins, flavonoids, tannins, among others.^[1–4]^ It is known that experiments with animal models, especially rodents, are essential for understanding the mechanisms that trigger diseases, in addition to discovering methods aimed at prevention and/or treatment.^[5]^

Rats and mice stand out for their small size, short biological cycle, lower maintenance cost, and similarity to humans, from anatomical points to common genes (95%),^[6]^ making it possible in the future for the results obtained from these experiments can be reproduced in human trials.^[5]^

However, in addition to animal studies, cell and in vitro experiments have been applied to mimic the human gastrointestinal tract (GIT) and show how biomolecules, especially proteins, behave when exposed to these physiological conditions.^[7–13]^

In this perspective, it is important to carry out systematic reviews focused on this theme to increase the accuracy of the results obtained in vivo tests. Besides, decreasing the risk of false-negative outcomes, improving the methodological quality of the experiments, among others.^[14]^ So, the registration of study protocols is considered an excellent tool in reducing the accumulation of research due to non-notification and reducing publication bias.^[15–18]^

Adopting in vitro test protocols is another way to obtain quick and adequate information regarding the application of biomolecules. In the scientific literature, there is a large availability of protocols in vitro, emphasizing the digestion and absorption systems. Among the applications, these systems can investigate the effects of cells or models that mimic biological conditions in humans.

Through these protocols, it is possible to generate important data, such as the application of the bioactive, since the main route of administration in vivo is oral. Several in vitro models are available to mimic the GIT, for example, models based on primary cells, monoculture or co-culture systems, and in vitro digestibility systems.^[19–21]^

Thus, validating the effectiveness of these protocols is essential as it mimics human biological conditions through in vitro studies decreasing the use of animals. Therefore, in compliance with international guidelines such as the 3R's program (reduction, refine and replace), which is against the unnecessary use of animals and encourages the minimization of this practice.^[22]^

Furthermore, according to Smith et al,^[23]^ protocol registration can help standardize clinical research. Thus, this proposed review protocol aims to elaborate on 2 systematic reviews to identify the analysis protocols used in animal, cellular, and in vitro standardized models to mimic digestive and absorptive processes of peptides and/or proteins.

## 2 Methods

### 2.1 Protocol and registration

This protocol was prepared according to the guidelines described by Preferred Reporting Items for Systematic Reviews and Meta-Analyzes Protocols (PRISMA-P).^[24]^ A 17-item checklist was used to improve the quality of the systematic review data. The protocol was registered with the International Prospective Register of Systematic Reviews (PROSPERO) on August 25, 2020 (protocol number: CRD42020198709), available in: https://www.crd.york.ac.uk/prospero/display_record.php?ID=CRD42020198709.

### 2.2 Eligibility criteria

Peer-reviewed journal articles that meet the eligibility criteria based on the study population, interventions, control, outcomes, and study design (PICOS)^[25]^ will be included in the review. There will be no restriction on the language and year of publication. Review studies and gray literature will not be included.

#### 2.2.1 Inclusion criteria

##### 2.2.1.1 Participants

For this review, original articles resulting from in vivo studies carried out with rats and mice of both sexes and varied ages (puppies, youngsters, adults, or the elderly) without the restriction of water or diet will be included, and also in cell and in vitro studies (gastrointestinal simulating fluids).

##### 2.2.1.2 Types of intervention

Studies in which the intervention group has been submitted to the administration of peptides, proteins, or gastrointestinal simulator fluids to mimic digestive and absorptive processes of peptides and proteins.

##### 2.2.1.3 Types of controls

Studies will be inserted that present the control group composed of animals, cells, or in vitro experiments without administration of peptides and/or proteins.

##### 2.2.1.4 Outcome measures

Studies that describe the model used to mimic protein digestion or absorption.

#### 2.2.2 Exclusion criteria

##### 2.2.2.1 Participants

Studies with models of digestion and absorption with other animals, or studies with computer simulations (in silico).

##### 2.2.2.2 Types of intervention

Studies that mimic the processes of digestion and absorption with the application of non-protein molecules associated with peptides and/or proteins will be excluded.

##### 2.2.2.3 Types of controls

Studies without a control group.

##### 2.2.2.4 Outcome measures

Articles that do not describe the protocol to simulate gastrointestinal conditions. Studies that do not have a schedule, time of experiment, frequency, dosages administered, concentration, and temperature will be excluded.

### 2.3 Information sources and literature search

Research strategies will be adopted based on keywords indexed in Medical Subject Headings (MeSH) (Tables 1 and 2).

**Table 1:** Keywords indexed in the Medical Subject Headings (MeSH) for the composition of the search equation strategies for gastrointestinal digestion.

KEY WORDS			
DigestionDigestibilityDigestion processBioavailabilityProteolysisHydrolysate	OralStomachGastricGastrointestinalGastrointestinal tractSmall intestinal	PeptidesProteinsProtein hydrolysate	AnimalMiceRatIn vitro

**Table 2:** Keywords indexed in the Medical Subject Headings (MeSH) for the composition of the search equation strategies for intestinal absorption.

KEY WORDS		
AbsorptionBioavailability	PeptidesProteins	AnimalMiceRatIn vitroCellCell cultureCaco-2

Two reviews will be elaborated on, one related to gastrointestinal digestion and the other about intestinal absorption. The equations will be defined considering the combinations of descriptors and their synonyms related to each review. The descriptors will be accompanied by the Boolean operators OR and/or AND.

The search strategies will be applied in the following electronic databases: PubMed; ScienceDirect; Scopus; Web of Science; Evidence portal, Virtual Health Library, and EMBASE. Two researchers will independently analyze the search keys (preliminary analysis) and will obtain the studies’ return found in this stage. The results will assist in the assembly of the definitive equation for each electronic database. All articles will be imported into the Rayyan application (version 0.1.0),^[26]^ the migration of articles to this platform will facilitate the removal of duplicate studies.

Our preliminary evaluation will be conducted based on the search for articles that mimic the digestion and absorption processes with peptides and/or proteins. For this purpose, 2 researchers, independently, will read the title and abstract respecting all eligibility criteria.

**Figure 1:**
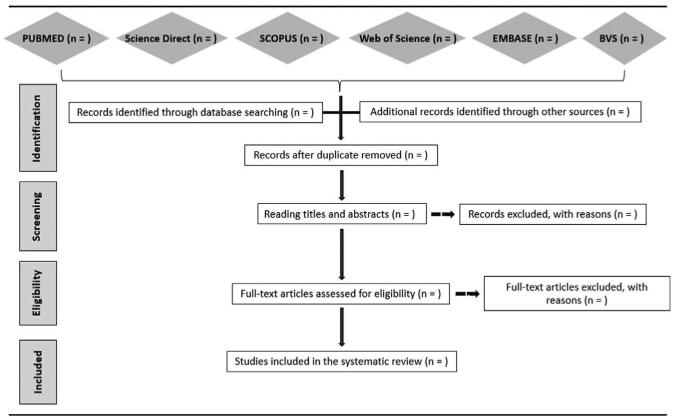
Article selection flowchart. Adapted from Preferred Reporting Items for Systematic Reviews.

The secondary evaluation will be carried out from the complete reading of the articles considered eligible for inclusion. It is noteworthy that emphasis will be placed on methods to identify analysis protocols that comply with the objectives of the systematic review (Fig. 1). Discrepancies will be resolved with the support of a third researcher. The references of the included articles will also be revised to identify those potentially eligible studies not found in the database search, considered a manual search. For the management of references, the Mendeley software^[27]^ will be applied.

### 2.4 Data extraction

Two reviewers will independently extract the data for each article. A spreadsheet will be created, and the data of interest for each review (gastrointestinal digestion and intestinal absorption) will be inserted. For this purpose, the Microsoft Excel Program will be used. The reviewers will be extract data related to the protocol adopted to simulate gastrointestinal processes (experiment time, frequency, dosages administered, concentration, and temperature) in all studies.

For animal studies will also be extracted – animal species, animal lineage, stage of life, sex; for cell studies – cell origin, the genetic background of cells (normal or cancerous), cell line types (intestinal epithelium); and for in vitro articles – composition of gastrointestinal simulator fluids, type of substance used, enzymes used, time and temperature of gastrointestinal processes. For any relevant data missing from the manuscripts, the reviewers will attempt contact with the study authors. If the necessary information is not obtained, the data will be excluded from the analysis and covered in the discussion section.

### 2.5 Risk of bias

Two reviewers will independently assess the risk of bias for each study entered. A third reviewer will resolve the discrepancies. For animal studies, the risk of bias assessment will be performed using the instrument of the Systematic Review Center for Experimentation with Laboratory Animals (SYRCLE).^[28]^ The Office of Health Assessment and Translation (OHAT) tool will be used to assess the risk of bias in cell studies (SERVICES, 2019).^[29]^ The reviewers will be previously trained and calibrated to ensure uniformity in the evaluation of the criteria.

### 2.6 Data analysis and synthesis

For both reviews, results summaries will be provided, and the protocols similarity in the simulation of digestion and/or absorption in vivo. The data will be presented in summary tables and in narrative forms to describe the characteristics of the included studies. These data will be structured with the type of protein used to simulate digestion and absorption, animal species and lineage, dose, type of administration, treatment time, and way of experimenting. Besides, considering other experimental conditions, including volume of simulator fluids, types of substances used in each stage of digestion and/or absorption, cultivation conditions, pH, time, and temperature, techniques and tools to study digestion and absorption in vivo and in vitro. It is noteworthy that no meta-analysis is applied for the type of study proposed.

## 3 Discussion

This protocol article aims to describe the development of 2 systematic reviews. In the first review, it is expected to identify the protocols related to protein digestion processes; in the second, the absorptive processes of peptides and/or proteins with emphasis on protocols that mimic these processes in humans involving studies with animals, cells, and in vitro (gastrointestinal simulating fluids).

From the studies, which will have the content and methodological quality critically evaluated, it will be possible to assist the scientific community in using models that can assess any protein for any purpose, as long as they need to analyze digestion and/or absorption processes.

Researchers highlight the bioactive potential of hydrolyzed proteins.^[30–32]^ Protein hydrolysis procedures involve chemical and enzymatic methods, among which changes in pH, temperature, and the use of enzymes can be highlighted.^[32]^ Therefore, it is crucial to develop studies to understand the behavior of proteins orally administered since they have better tolerance in administration.^[33]^

However, some drugs have reduced bioavailability and bioaccessibility. It is noteworthy that parts of these drugs are peptides and proteins. However, there are limitations in applying of proteins due to their low solubility, low permeability, rapid degradation in the GIT, and the inability to permeate the mucus barrier.^[34]^ Therefore, it is essential to carry out studies to understand the behavior of plant protein administration in the GIT, as their functionality can be modified.^[35]^

Based on an in vitro system and cell cultures, the methods are frequently used for simulation studies of the gastrointestinal environment. Through them, it is possible to mimic exclusive characteristics of GIT, which helps to understand various effects, in particular, in the identification of peptides and drugs that can be absorbed by intestinal epithelial cells.^[36–38]^ Although there is a great impact of in vitro studies on the efficacy, stability, and biological activity, the availability of such peptides and proteins for oral delivery in humans must also be verified in vivo experiments.^[38,39]^

Several types of research with animal models, mainly rodents, have shown that the degree of genetic similarity with human beings allows extrapolating the scientific results obtained for potential effects of treatment in humans.^[6]^ In this sense, numerous models are available to mimic the GIT and, and show how these biomolecules, especially proteins, behave when exposed to this scenario/environment.^[40,41]^

Bringing together studies that adopt these protocols is essential since a vast series of protocols mimic the gastrointestinal processes of different food components can be found in the literature. However, there is a scarcity of systematic studies of models that mimic the digestion and absorption of proteins.

Thus, the reviews in question intend to share with the scientific community which protocols have been used to simulate the digestion and absorption of proteins in models in vivo, with cells, or in vitro (gastrointestinal simulating fluids). These studies are promising tools because they can help understand how bioactive proteins, after digestion and absorption, manage obesity and its comorbidities. It will be important to guide researchers on the feasibility of applying new bioactive proteins to humans.

## Author contributions

**Conceptualization:** Anna Beatriz Santana Luz, Rafael Oliveira de Araújo Costa, Grasiela Piuvezam, Ana Heloneida de Araújo Morais.

**Data curation:** Anna Beatriz Santana Luz, Rafael Oliveira de Araújo Costa, Gidyenne Christine Bandeira Silva de Medeiros, Grasiela Piuvezam, Ana Heloneida de Araújo morais.

**Formal analysis:** Anna Beatriz Santana Luz, Rafael Oliveira de Araújo Costa, Gidyenne Christine Bandeira Silva de Medeiros, Grasiela Piuvezam, Ana Heloneida de Araújo morais.

**Funding acquisition:** Ana Heloneida de Araújo morais.

**Investigation:** Anna Beatriz Santana Luz, Rafael Oliveira de Araújo Costa, Grasiela Piuvezam, Gidyenne Christine Bandeira Silva de Medeiros.

**Methodology:** Anna Beatriz Santana Luz, Rafael Oliveira de Araújo Costa, Grasiela Piuvezam, Gidyenne Christine Bandeira Silva de Medeiros.

**Project administration:** Grasiela Piuvezam, Ana Heloneida de Araújo Morais.

**Supervision:** Grasiela Piuvezam, Gidyenne Christine Bandeira Silva de Medeiros, Ana Heloneida de Araújo Morais.

**Validation:** Thais Souza Passos, Ana Heloneida de Araújo morais.

**Writing – original draft:** Anna Beatriz Santana Luz, Rafael Oliveira de Araújo Costa, Gidyenne Christine Bandeira Silva de Medeiros, Thais Souza Passos.

**Writing – review & editing:** Anna Beatriz Santana Luz, Rafael Oliveira de Araújo Costa, Grasiela Piuvezam, Gidyenne Christine Bandeira Silva de Medeiros, Thais Souza Passos, Ana Heloneida de Araújo Morais.
